# On the epidemiology of *Plasmodium vivax* malaria: past and present with special reference to the former USSR

**DOI:** 10.1186/s12936-018-2495-y

**Published:** 2018-10-04

**Authors:** Anatoly V. Kondrashin, Lola F. Morozova, Ekaterina V. Stepanova, Natalia A. Turbabina, Maria S. Maksimova, Evgeny N. Morozov

**Affiliations:** 1 Sechenov University, Moscow, Russian Federation; 2 Department of Tropical, Parasitic Diseases and Disinfectology, Russian Medical Academy of Continuous Professional Education, Moscow, Russian Federation

**Keywords:** *Plasmodium vivax*, Elimination, Epidemiology, Severe malaria, Asymptomatic malaria, Primaquine, G6PD deficiency, Long incubation

## Abstract

Presently, many malaria-endemic countries in the world are transitioning towards malaria elimination. Out of the 105 countries with ongoing malaria transmission, 10 countries are classified as being in the pre-elimination phase of malaria control, and 9 countries are in the malaria elimination stage, whereas 7 countries are classified as being in the prevention of introduction phase. Between 2000 and 2015, 17 countries eliminated malaria (i.e., attained zero indigenous cases for 3 years or more). Seven countries were certified by the WHO as having successfully eliminated malaria. The purpose of this review was to analyse the epidemiological characteristics of vivax malaria during the various stages of malaria eradication (elimination) programmes in different countries in the past and present. Experiences of the republics of the former USSR with malaria are interesting, particularly since the data overwhelmingly were published in Russian and might not be known to western readers. Among the most important characteristics of *Plasmodium vivax* epidemiology at present are changes in the ratio of the short-incubation *P. vivax* to long-incubation *P. vivax,* the incidence of severe *P. vivax* cases, the increased numbers of asymptomatic *P. vivax* cases, the reduced response to anti-malarials and a few others. Various factors contributing towards the peculiarities of *P. vivax* epidemiology are discussed.

**Electronic supplementary material:**

The online version of this article (10.1186/s12936-018-2495-y) contains supplementary material, which is available to authorized users.

## Background

Many malaria-endemic countries are transitioning towards malaria elimination. At present, out of 105 countries with ongoing malaria transmission, 10 countries are classified as being in the pre-elimination phase of malaria control, and 9 countries are in malaria elimination stage, whereas 7 countries are classified as being in the prevention of introduction phase. Between 2000 and 2015, 17 countries eliminated malaria (i.e., attained zero indigenous cases for 3 years or more) [1].

Globally, more countries are moving towards elimination; in 2016, 44 countries reported fewer than 10,000 malaria cases, up from 37 countries in 2010. Kyrgyzstan and Sri Lanka were certified by WHO as malaria-free in 2016, thus contributing to the total of 7 countries certified during the last few years. In 2016, WHO identified 21 countries with the potential to eliminate malaria by the year 2020 [2]. Experiences in malaria eradication (in the past) and elimination (at present) have revealed that in the countries with local transmission of both falciparum and vivax malaria, the successful implementation of various anti-malaria measures results first in the disappearance of *Plasmodium falciparum,* followed by its elimination. This phenomenon is due to the biological characteristics of *Plasmodium vivax*, particularly the presence of the hypnozoites responsible for relapses of this species. In addition, various genotypes of *P. vivax* demonstrate differences in the primary appearances of clinical signs of the disease, thus dividing the parasite populations into 2 groups: *P. vivax* with a short or long incubation period. On average, as shown from experience, the elimination of *P. vivax* foci can be achieved, but not in fewer than 3 years, compared with the elimination of *P. falciparum*, which can be achieved in 1 year [3].

The purpose of this review is to analyse the epidemiological characteristics of *P. vivax* during the various stages of malaria eradication (elimination) programmes in different countries in the past and present. The experiences of republics of the former USSR with malaria are interesting, particularly because an overwhelming amount of data was published in Russian and might not be known to western readers.

Among the most important characteristics of *P. vivax* epidemiology at present are changes in the ratio of *P. vivax* with short incubation to *P. vivax* with long incubation, an increased incidence of severe *P. vivax* cases, increased numbers of asymptomatic *P. vivax* cases, reduced response to anti-malarials and a few others.

## Changed short-to-long incubation ratio in vivax malaria

Populations of *P. vivax* with long incubation were originally confined to areas with a temperate climate, such as northern, eastern and central Europe, northern parts of Asia and America, whereas *P. vivax* with short incubation has overwhelmingly been distributed in areas with sub-tropical and tropical climate. However, during the last few decades, a well-established trend of proliferation of *P. vivax* with long incubation is occurring to the south. Such events could have implications.

**Fig. 1:**
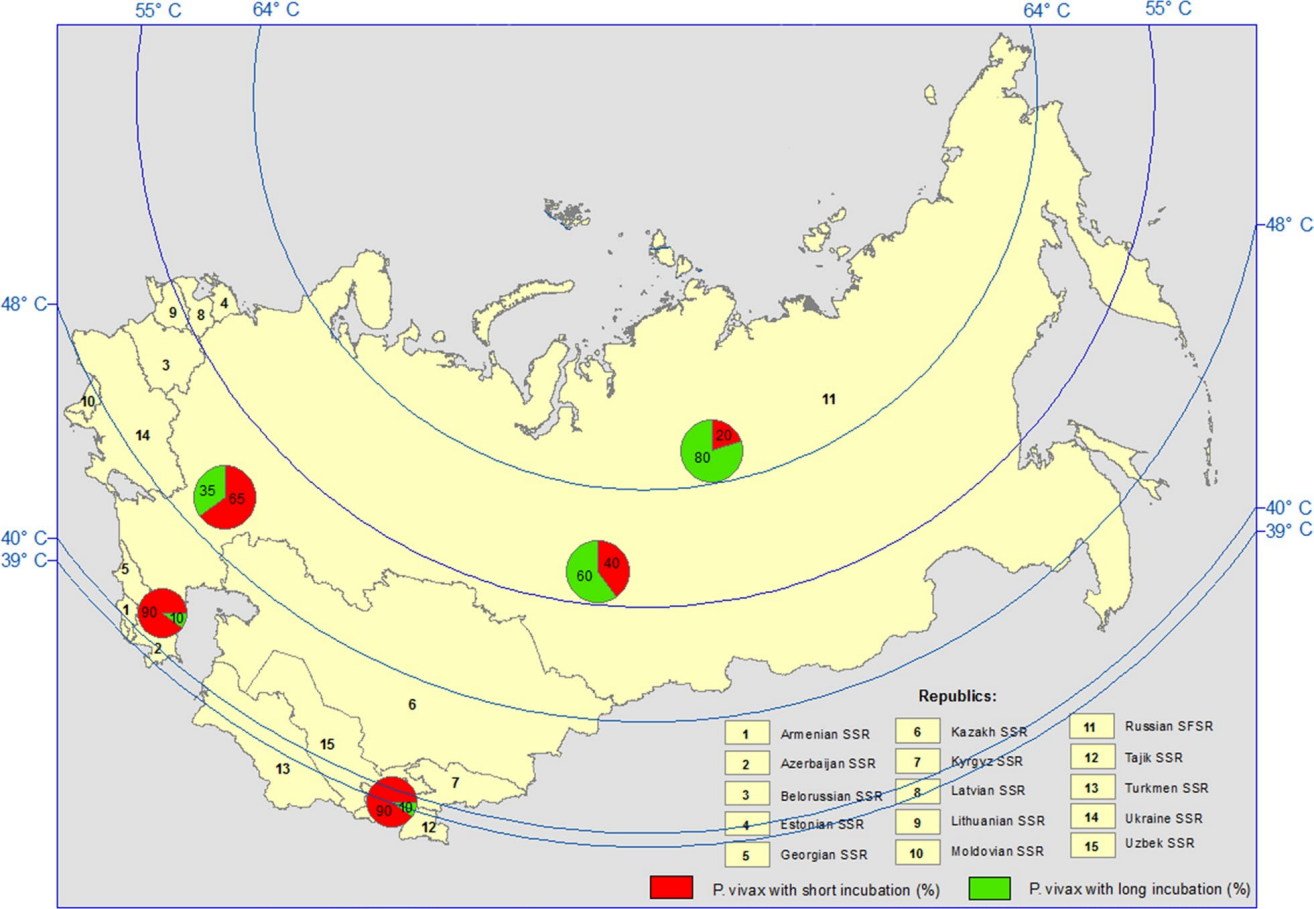
Ratio of *Plasmodium vivax* short incubation to long incubation

For example, prior to the launch of large-scale malaria control activities in the area of the European part of the former USSR, *P. vivax* with long incubation constituted approximately 70–80% of the total malaria cases. Primary clinical manifestations usually occurred at 8- to 14-month intervals following contraction of infection. However, in the southern parts of the country, the proportion of vivax malaria with long incubation accounted for approximately 10% of the total malaria cases, with the remaining cases being vivax malaria with short incubation [4] (Fig. 1).

With the implementation of countrywide activities of the national malaria eradication campaign during the 1950s and 1960s, the intensity of malaria transmission was considerably reduced, which resulted in the alteration of the ratio exemplified by the malaria situation in the Republic of Azerbaijan.

During the 1940s and 1950s, *P. vivax* with short incubation was the predominant species in the Republic of Azerbaijan, accounting for up to 90% of the total malaria cases. No cases of *P. falciparum* were registered at all. By the end of the 1960s, the territory of the Republic was free of malaria except for the continued transmission in a few residual foci of vivax malaria.

**Fig. 2:**
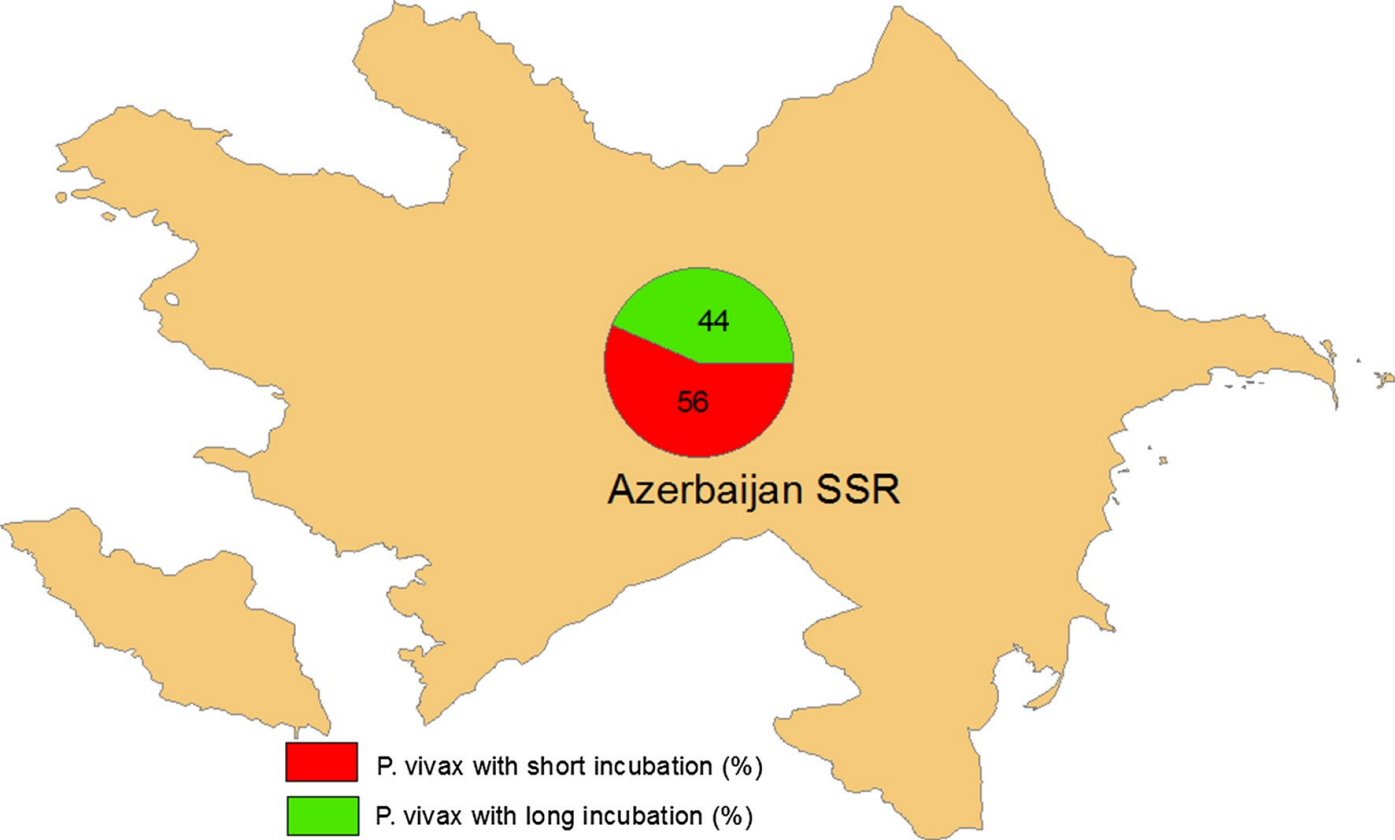
Ratio of *Plasmodium vivax* with short incubation to that with long incubation (Republic of Azerbaijan 1980s and 1990s)

Due to the interaction of various factors, a large-scale epidemic of vivax malaria occurred in the plains of the Republic of Azerbaijan in the 1970s. During the course of the epidemic, the number of primary clinical manifestations on the eve of the start of local malaria transmission (month of May) dramatically increased, suggesting the occurrence of malaria with long incubation. Such cases constituted 32–36% of the total number of cases detected during the years 1971–1973 [5] (Fig. 2).

Results of studies in 3 *P. vivax* residual malaria foci in the Geok-Chai District of Azerbaijan in 1980–1983 revealed that the fraction of *P. vivax* with long incubation constituted 49% of the cases, and some cases were detected up to 28 months after contraction of infection [6].

**Fig. 3:**
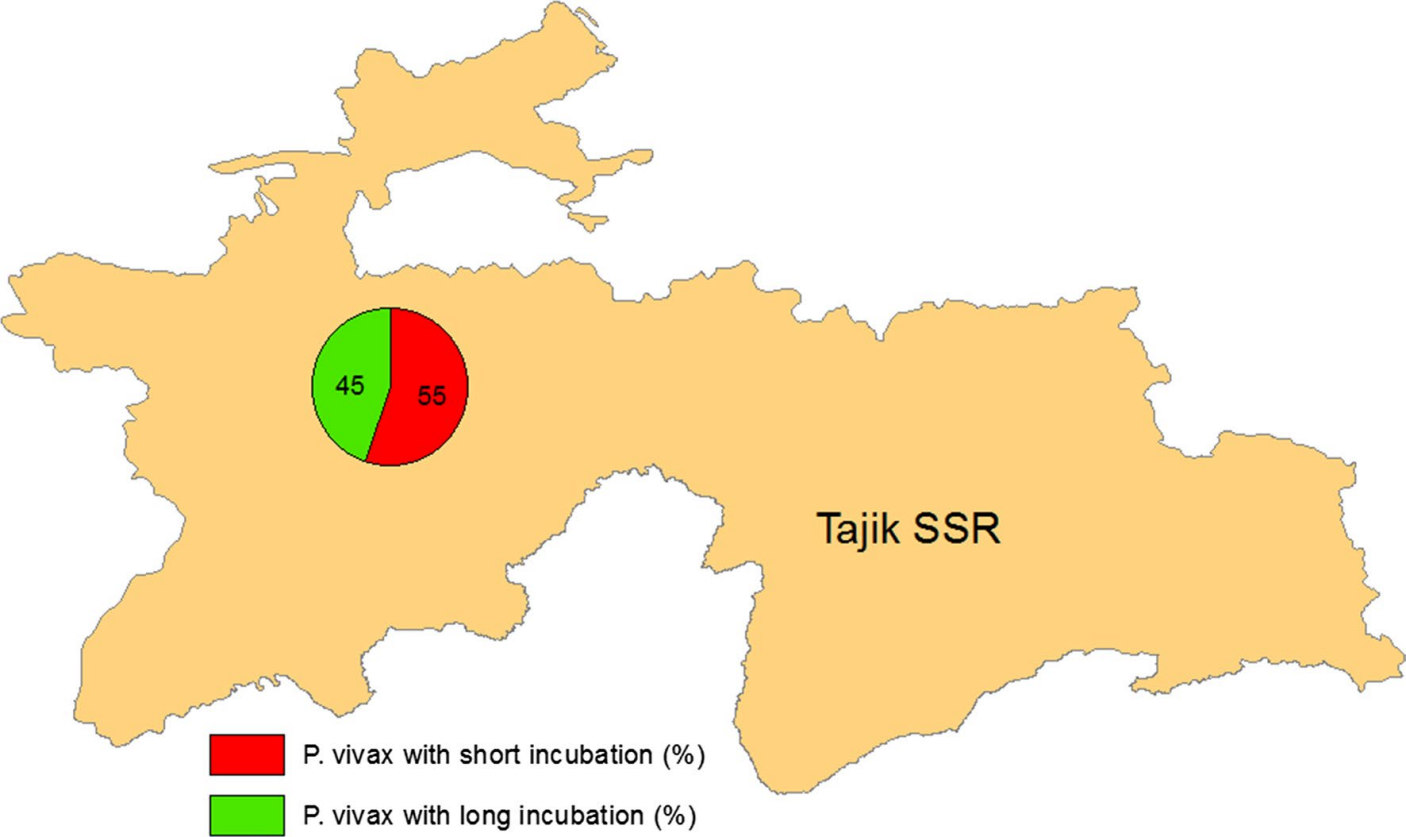
Ratio of *Plasmodium vivax* short incubation to long incubation (Republic of Tajikistan 1990 and 2000s)

In the Republic of Tajikistan, *P. vivax* cases with short incubation constituted more than 95% of the total cases during the 1940s and 1950s (Fig. 3).

However, in 1988–1990, in the Punj district bordering Afghanistan, 45% of all detected cases were classified as *P. vivax* cases with long incubation [7]. In other border areas with Afghanistan, the proportion of cases with long incubation constituted 55% [8]. Among the imported cases of *P. vivax* in Soviet servicemen from Northern Afghanistan during the 1980s, 54% were *P. vivax* cases with long incubation. The primary clinical manifestations of these cases were registered from 6 to 38 months after contraction of infection [9].

Reports from other parts of the world, particularly from the Indian continent and Southeast Asia have further confirmed that, at present, *P. vivax* with long incubation is no longer a legacy of only those countries with temperate climate [10, 11]. Now, *P. vivax* phenotypes with long incubation are believed to be more widespread and more prevalent than previously thought [12]. For example, in China, where *P. vivax* is a predominant malaria species, the ratio of short to long incubation is approximately 1:1 [13, 14].

As in the past, North Korea continues to be the stronghold of *P. vivax* strains, with more than 70% of the malaria cases being *P. vivax* with long incubation [15].

In India, studies in the western territories (Gujarat State) revealed the presence of *P. vivax* malaria with long incubation [16]. The presence of local strains of vivax malaria with long incubation in Delhi (north/central India) was confirmed during studies in 1988–1993 [10]. The existence of *P. vivax* with long incubation in Central India was reconfirmed by the results of studies carried out in Aligarh [17]. The results of genotyping of *P. vivax* carried out in the eastern part of the country (Kolkata) revealed the presence of locally transmitted infection with primarily long incubation, which occurred 81% of the time compared with short incubation at 19% [11].

The results of these observations have important bearings on the organization and implementation of anti-malarial activities. Unlike the case of *P. falciparum*, elimination of vivax malaria foci cannot be achieved during a 1-year period, thus necessitating the continuation of interventions and observations for at least 3 consecutive years and also increasing the cost of the operations. Another important peculiarity of the epidemiology of *P. vivax* at present is the increase in the number of reported cases of severe infection.

## Incidence of severe *Plasmodium vivax* cases in the former USSR

**Fig. 4:**
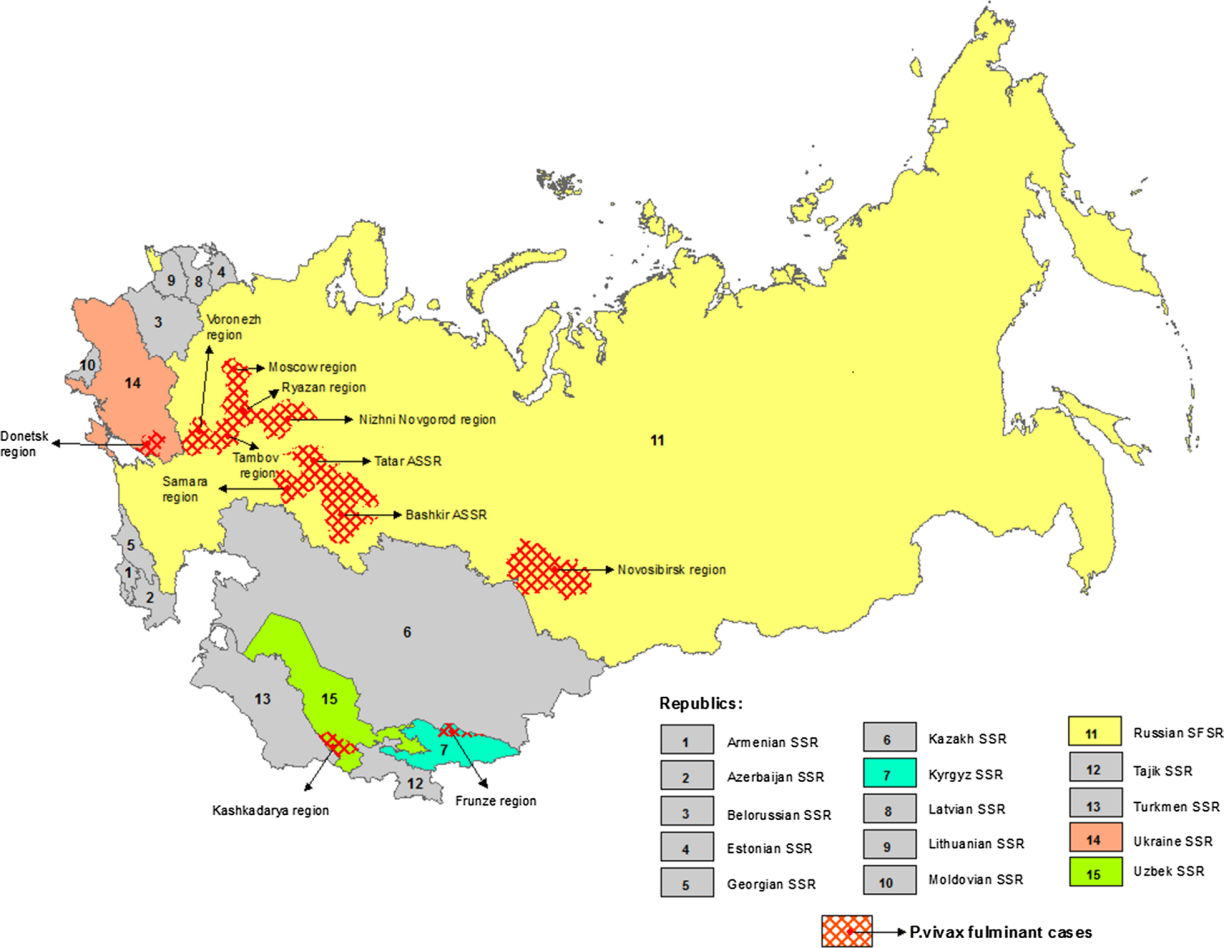
Registered *Plasmodium vivax* fulminant cases in the former USSR

In the former USSR, prior to malaria elimination in the country, malaria was one of the major health problems (see Additional file 1), and complicated cases of *P. vivax* were registered quite regularly [18]. During the 1930s and 1940s, an epidemic of so-called *P. vivax* ‘fulminant malaria’ known for causing death, occurred on the territory of the European part of the USSR and in Kyrgyzstan and Uzbekistan (Fig. 4). Fulminant malaria was common among both children and adolescents and rather rare among adults. Normally, cases of fulminant malaria were reported during the spring and summer months, coinciding with primary manifestations of the disease or following 2–3 relapses in the case of *P. vivax* with long incubation. Death could occur within 2–3 h [18] (see Additional File 2). Russian clinicians believed that such forms of vivax malaria were not only species-specific but were also related to the health state of the infected individual in conjunction with a very poor socio-economic environment [19].

Detailed descriptions of severe cases of vivax malaria in the USSR have been presented in monographs and various journals in Russian. The opinion of Soviet clinicians was that the major cause of severe disease was due to ‘secondary hypochrome’ anaemia from prolonged high parasitaemia [20]. Modern explanations of the phenomenon agree with this statement. High parasitaemia in *P. falciparum* (approximately 100,000 parasites/µl) is less pernicious than parasitaemia of 10,000–20,000 parasites/µl in *P. vivax,* as the latter persists in the organism considerably longer than in cases of *P. falciparum* [21].

Cases of comatose *P. vivax* were reported in approximately 5% of the total comatose malaria cases and exclusively during malaria epidemics [18]. Rupture of the spleen was one of the most serious complications in *P. vivax,* leading to acute abdominal pain accompanied by a sharp reduction in blood pressure, internal haemorrhage and shock [8, 20]. Other manifestations (1–2% of total complications) were haemoglobinuria, acute nephritis, hypertension, haematuria, albuminuria, and several other rare syndromes [18].

## Present increase of severe vivax malaria in the world

An increase in the numbers of severe cases and even cases of deaths due to *P. vivax* in the world marked the beginning of the 21st Century and coincided with the commencement of national programmes of malaria elimination. This increase resulted in the appearance of a number of publications on the subject in different parts of the world [22–25].

As could be expected, more than 40% of all publications on *P. vivax* come from India, the country with the world’s largest national malaria control programme and where the annual number of *P. vivax* cases constitutes 47% of all malaria cases [26]. Serial cases of severe *P. vivax* have been reported in the USA, Indonesia and Pakistan. Sporadic cases have also been reported in Laos, Cambodia, Thailand, China, DPR Korea, Bangladesh, Afghanistan, countries of the Middle East and the African Horn, Madagascar, Brazil, and Papua New Guinea [27].

Among the major clinical signs of complicated vivax malaria, severe thrombocytopaenia (< 50,000 cub.mm) is the most common manifestation. The association of severe thrombocytopaenia with acute anaemia can result in death of the malaria patient [28]. Acute anaemia in vivax malaria constitutes a high mortality risk for young children and pregnant women, with a reported case fatality rate of 0.3% [22, 25].

Overall, severe illness with acute infection includes lung injury with respiratory distress, kidney injury with renal dysfunction, hepatic dysfunction and jaundice, seizures/delirium/coma, severe thrombocytopaenia or circulatory collapse [29]. The severity of the syndromes varied widely among the different geographical areas, with severe anaemia being most prominent in areas of high transmission, frequent relapses and chloroquine resistance [24, 25].

Sporadic cases of complicated vivax malaria described in India and Brazil revealed a few other syndromes such as jaundice, haemoglobinuria, seizures, and pulmonary oedema. A combination of syndromes such as jaundice, thrombocytopaenia, anaemia, renal dysfunction, respiratory distress, and cerebral malaria resulted in the death of patients in India [30].

*Plasmodium vivax*-related anaemia is a high risk for children. Thus, in malaria-endemic territories of Indonesia, approximately 25% of the total inpatients with apparent anaemia are young children [31]. Severe *P. vivax*-related anaemia among hospitalized children in Papua (Indonesia) was the cause of death at the level of 10.3 per thousand cases [22].

The details of *P. vivax*-related anaemia in pregnant women were determined in Russia. The most frequent complications were premature delivery, abortions, prolonged course of the disease, and specially increased frequency of relapses towards the end of pregnancy and immediately after delivery [18]. The risk of severe anaemia in pregnant women affected by *P. vivax* is double that of non-pregnant women with vivax malaria [32]. Studies undertaken in Peru in areas with relatively low levels of vivax malaria transmission revealed that even a single attack of infection during the first trimester of pregnancy increased the risk of spontaneous abortion by four times [33].

The situation has further been aggravated by the exclusion of pregnant women and young infants/children from the use of a gametocytocidal drug (primaquine) in *P. vivax* infection, thus contributing to local malaria transmission being an important source of infection [21].

Severe *P. vivax* cases can be reported in situations with very low local transmission. Two cases of severe vivax malaria were described in such a setting in the Russian Federation. One was an introduced case in Volgograd city in 1998 in a 62-year-old man. Malaria was laboratory-confirmed only on the 10th day after primary clinical manifestations. Detailed clinical examination of the patient revealed the presence of severe anaemia and liver cirrhosis. In spite of the anti-malaria treatment undertaken, the patient died [34]. Another introduced case occurred in Moscow in 2001 in a man 35 years of age. The patient was admitted to the hospital with acute spleen pain and haemorrhage which necessitated a splenectomy due to rupture; subsequently, parasites of *P. vivax* were detected in the patient’s blood [34].

## Clinical and parasitological manifestations of *Plasmodium vivax* in the elimination stage

The intensity of reproduction of the malaria parasite determines both the clinical course of the infection and the stability of the epidemiological process. Evidence exists of differences in clinical and parasitological manifestations during the increase/decrease of malaria incidence, corresponding to various periods of malaria season [35].

Duchanina [36] described a mild clinical course of *P. vivax* infection with long incubation during the final stages of malaria eradication in the former USSR in comparison with progression in the pre-eradication period. This phenomenon was attributed to contraction of malaria from a mosquito infected by a small number of sporozoites and the absence of repeated infection by mosquito [36].

A similar situation was found in Tajikistan and in Azerbaijan in the course of the final stage of containment of a malaria epidemic during the 1980s. A marked decrease occurred in the clinical manifestations, along with low parasitaemia among cases with long incubation, which was accompanied by an increase in the number of asymptomatic carriers [37, 38]. A mild course of *P. vivax* infection was observed among 92 children under 14 years of age in one study in the Punj district of southern Tadjikistan in 1988–1990. Infected children with body temperatures of 37.2–38.0 °C did not have any complaints and attended school classes [39]. Thermometry surveys among women engaged in the cotton harvesting in Azerbaijan detected appreciable numbers of malaria patients without complaints while their body temperature was 38 °C (Kondrashin, pers. comm.).

In Azerbaijan, laboratory examination of blood slides taken from *P. vivax* patients with long incubation in the residual foci demonstrated lower parasite densities in comparison with the density of parasites in the blood films taken from patients with short incubation [40]. Data from the National Malaria Reference Centre in Baku, Azerbaijan, showed a decrease in parasite densities of *P. vivax* was associated with a decrease in the intensity of malaria transmission. In 1974–1976, at the height of malaria outbreak, the proportion of positive blood slides for *P. vivax* with a low density of parasites was approximately 20%, while this proportion was 37% at the beginning of the 1980s following a reduction in malaria transmission intensity [41].

An implication of low clinical manifestations and parasitaemia in malaria cases following a reduction in the intensity of malaria transmission is difficult in the case of detection; low clinical manifestation prolongs the time for laboratory confirmation of malaria diagnosis and necessitates the deployment of much more sensitive and costlier diagnostic methods such as PCR, etc. Reduced clinical manifestation also suggests that long-incubation *P. vivax* is more prone to survive in the form of asymptomatic case.

## Factors facilitating incidences of severe vivax malaria

Unification of the definition of ‘severe *P. vivax* case’ recently by the WHO has facilitated the reporting of such cases by the personnel of health treatment facilities [42]. This was done through the modification of the definition of ‘severe *P. falciparum* case’. Severe vivax malaria is defined as it is for falciparum malaria but with no parasite density threshold [42]. For example, the results of observations in one hospital in India revealed that, among the vivax malaria patients, 15% were reported cases of complicated disease [23].

## Comparative epidemiological role of *Plasmodium vivax* with short and long incubation

The results of genotyping of *P. vivax* parasites (presumably relapses) carried out in the vicinity of Kolkata (India) revealed active transmission of both short- and long-incubation phenotypes [11]. Furthermore, the proportion of true relapses was 60%, whereas that of re-infection constituted 40%. Detailed analysis revealed that relapses due to short incubation were genetically homogenous in 69% of the cases, whereas the remaining relapses were genetically heterogeneous [11]. Implications of these findings are that re-infection due to heterogeneous strains, as a rule, result in a more severe disease course [20]. The same phenomenon is observed in a case of re-infection by a malaria parasite other than *P. vivax*, particularly re-infection by *P. falciparum* [20].

**Fig. 5:**
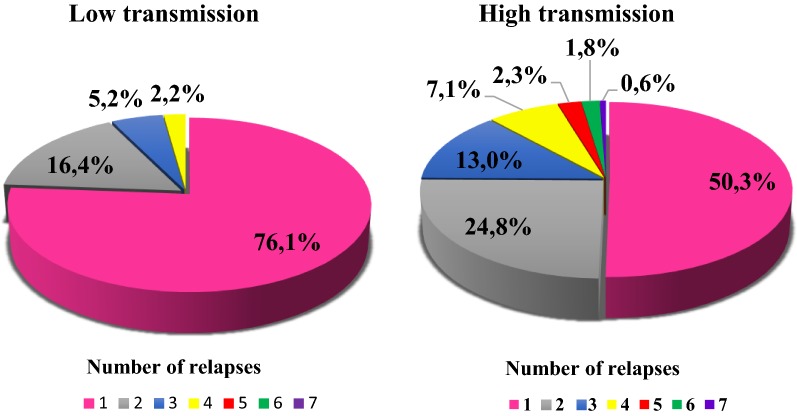
Incidence of relapses of vivax malaria with long incubation in foci with various level of transmission [43]

One of the peculiarities of the epidemiology of vivax malaria with long incubation in the former USSR was the modulation of the number of relapses depending on the transmission level of infection (Fig. 5).

**Fig. 6:**
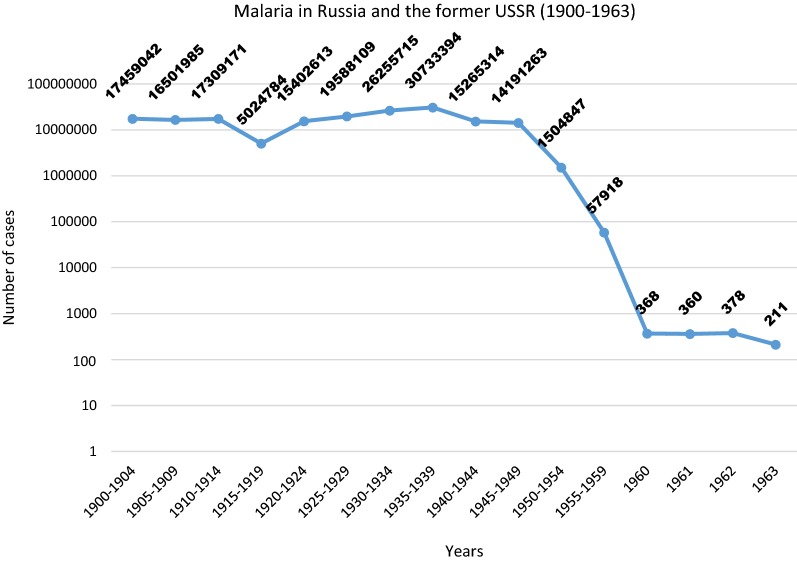
Malaria in Russia and the former USSR (1900–1963)

The frequency of relapses depending on the transmission level has an important bearing on the clinical course of vivax malaria. Strains with short intervals between relapses, as in the case of the Chesson strain and strains with long incubation, have negative effects on the haematological state of an infected individual in comparison with *P. vivax* strains with a long interval between relapses. The cause of such a phenomenon is that the appearance of short relapses overrides the ability of the blood system to return to its normal function following previous attacks of the disease. Such a relapse results in an absolute reduction in the red blood cell mass due to the greater removal of uninfected red blood cells [32] (Fig. 6).

The main difference between *P. vivax* primary manifestations and relapse is in the abrupt appearance of malaria syndromes in the case of the latter. At the same time, the level of parasitaemia is considerably higher compared with primary clinical manifestations. The level of parasitaemia could reach 5000 and even 10,000 parasites/ml compared with 200–500 parasites. However, the patient feels much better than during the primary manifestations due to the development of some level of immunity. Importantly, along with the increased density of parasites, a parallel augmentation occurs in the density of gametocytes. The results of one study in Georgia (in the former USSR) during the 1950s and1960s revealed that the density of gametocytes during *P. vivax* relapse was two times higher than during the primary manifestations of the disease [44]. Thus, the epidemiological role of *P. vivax* relapses could be seen in the enhancement of malaria transmission due to the higher density of gametocytes on the one hand and the potential increase of asymptomatic malaria on the other hand.

## Role of relapse pattern in the epidemiology of vivax malaria

Various strains of *P. vivax* differ in their innate ability to relapse. Thus, the ‘Madagascar’ strain was known for its ability to relapse in approximately 80% of cases in comparison with the ‘Holland’ strain, which relapsed in only 10% of the cases [3]. The relapse rate in the ‘Chesson’-like strains distributed in the area of the Western Pacific and Southeast Asia is even higher (more than 80%) [45]. On the other hand, *P. vivax* in the area of the sub-Indian continent (India, Nepal, Sri Lanka) historically is known for its relatively low ability to produce relapse [46]. In India, during the 1960s, the maximum level of relapses in local *P. vivax* was approximately 40% [47]. The dynamics of the innate ability of Indian strains of *P. vivax* to relapse are presented in Table 1.

**Table 1:** Dynamics of the innate ability to produce relapse and its frequency after 5-day treatment with primaquine in India (1960–2012)

Years	Source	Innate ability %	Relapse rate after 5-day treatment with primaquine
1960	Basavaraj [47]	40.00	5.75
1990	Sharma et al. [48]	40.00	2.60
1996	Srivastava et al. [16]	16.00–27.00	2.50–5.80
1998	Gogtay et al. [49]	8.30	3.00
1998	Adak et al. [10]	23.00–44.00	NA
2001 (a)^*^	Adak et al. [50]	40.00	27.00–30.00
2001 (b)^*^	Adak et al. [50]	21.80	19.80
2002	Yadav et al. [51]	8.60	5.70
2012	Kim et al. [11]	31.00	27.00

*Two different sites

During the appraised period, the innate rate of relapse hardly exceeded 40%, and in some areas, it was even much lower. The efficacy of a 5-day treatment with primaquine, particularly in conjunction with the use of insecticides on a large scale, was quite efficacious both from an operational and epidemiological point of view. However, the situation began to change the beginning of the 21st Century, when the efficacy of a 5-day treatment practically reached zero [51]. In spite of evident failing efficacy of a 5-day treatment with primaquine, the use of primaquine continued to be the official method of anti-relapse treatment recommended by the national malaria control programme in India until 2007. The replacement of the use of the 5-day treatment with the standard 14-day treatment took place only in 2008. The introduction on a national scale of the 14-day treatment was accompanied by several operational problems, with the very low level of compliance by the affected population being a significant problem [52]. The result was thousands of improperly treated people, who potentially were contributing to (a) the enhancement of local *P. vivax* transmission; (b) an increased number of severe cases; and, (c) an increased number of asymptomatic cases [52].

The malariological situation likely was further aggravated by the replacement of local strains of *P. vivax* with a low ability to relapse with strains with a high ability that was facilitated by large-scale, uncontrolled population movement from different malaria-endemic countries, including Papua New Guinea, Indonesia, Myanmar, Thailand, Afghanistan, and Pakistan. A probable mechanism of strain replacement might be the recombination of the various genotypes of the parasite, contributing to the biological diversity of the species [12].

## Role of asymptomatic infection in the malaria elimination process

Malaria infection in the form of asymptomatic parasitaemia occurs quite frequently in the world. In the former USSR, prior to the launch of a national malaria eradication programme, the prevalence of asymptomatic parasite carriers constituted 10–18% of all detected malaria cases during mass surveys of the population, particularly on the eve of the beginning of malaria transmission season in May and June [53].

In the sub-tropical Massali district of Azerbaijan during the final stage of malaria eradication, mass blood surveys in 2 active malaria foci in 1960 revealed that the prevalence of asymptomatic *P. vivax* cases was 23.7% of the total number of cases in one village and 56.2% in another village. The majority of detected carriers were children under 14 years of age [54].

In tropical areas, the frequency of asymptomatic *P. vivax* varies from 30 to 50% among the children and to a somewhat lesser extent among adults. Thus, a large proportion of asymptomatic infections of both *P. falciparum* and *P. vivax,* with low and sub-microscopic parasite densities, was found in the low-transmission setting of Temotu Province, Solomon Islands, in 2008 [55]. Only 17.8% of *P. falciparum*- and 2.9% *P. vivax*-infected subjects were febrile at the time of the survey. A significant proportion of both infections detected by microscopy showed a parasite density below 100 parasites/µl. An age correlation accounted for the proportion of low parasite density for *P. vivax* only [55].

In the Bandarban District of Chittagong Hill Tracts of southeastern Bangladesh, a large proportion of asymptomatic *P. falciparum* and *P. vivax* infections were found by microscopic and PCR examination. The proportion of asymptomatic infections among the confirmed PCR cases was 77%. Significantly more asymptomatic cases were recorded among the patients older than 15 years of age, whereas prevalence and parasite density were significantly higher in patients younger than 15 years of age [56]. A similar pattern was noted among the imported cases from Africa and Asia to the Russian Federation [57].

The possibility of transmission of malaria infection by *Anopheles* mosquitoes feeding on an infected human with a very low density of parasites in the peripheral blood was experimentally demonstrated in Russia as early as the end of the 1930s and beginning in the 1940s [58, 59]. At present, individuals with very low parasitaemia and/or with asymptomatic malaria provide an important reservoir for malaria transmission and smear-positive asymptomatic cases are more infectious to mosquitoes than those with sub-microscopic infection [60].

The genesis of asymptomatic malaria is largely of an immunological nature. The intensity of parasitaemia progressively decreases due to the active formation of anti-parasite complex immunity, whereas pyrogenic thresholds prevent malaria patients from undergoing malaria paroxysms under conditions of reduced density of malaria parasites. In other words, asymptomatic malaria disease is a consequence of infection experienced sometime in the past. The phenomenon of asymptomatic malaria is a potential threat to the infected person and to the community as well [57].

Asymptomatic malaria in an infected person is a good example of a well-balanced status between the host and parasite. However, this balance can change in favour of the latter due to various reasons (re-infection, environmental changes, reduction of immunity, etc.). Such disruption of the balance can occur at any time as the longevity of asymptomatic infection varies quite widely, and the density might be very low. In *Plasmodium malariae*, for example, asymptomatic infection can last for many years. Epidemiologically, the patient with malaria could possibly lead to the patient with asymptomatic malaria infecting a recipient (transfusion malaria). In the case of reduced immunity in the patient with asymptomatic malaria, the disease might return in a quite severe clinical form [57].

As far as the communities are concerned, asymptomatic malaria cases that harbour infective gametocytes are quite efficient in the maintenance of malaria transmission and are largely responsible for the persistence of malaria foci [61].

Various factors and conditions might facilitate the development of asymptomatic malaria. A broad range of factors associated with the development of asymptomatic malaria was recently reviewed [61]. Among the factors listed were infection prevalence, mosquito infectivity, parasite density, strain diversity, and many others. The role of factors such as polymorphism of immunologically relevant genes and genetic disorders (G6PD deficiency, β-thalassaemia, etc.), malaria infections in association with different parasitic diseases and infections, and others was also mentioned [61].

## Role of G6PD deficiency in asymptomatic vivax malaria

G6PD deficiency is a common genetic disorder in humans. This deficiency is prevalent throughout Africa, Asia, Southeast Asia and parts of South America, where malaria is endemic [62]. At present, the prevailing hypothesis is that G6PD deficiency confers protection from severe malaria disease caused by *P. falciparum*. The basis of this hypothesis was the results of several population-based studies indicating more light clinical manifestations of the disease among individuals with the G6PD deficiency accompanied by low parasitaemia [63–66]. Thus, a mild course of *P. falciparum* infection potentially results in asymptomatic malaria.

The relevance of this hypothesis to *P. vivax* was tested in Azerbaijan during the epidemic in 1970 to 1973. Epidemiological studies undertaken in the Republic found the G6PD deficiency present among local population at various levels (from 2.8% to 38.7%, with an average of 10.0%) [67]. This deficiency was highest among the people of the Geok-Chai District (on average 15.4%). The aim of studies undertaken in the Geok-Chai District was to establish whether the carriers of the deficient gene had a protective advantage against contracting vivax malaria. The studies were carried out in a group of villages with a total population of approximately 8000 people and with a malaria prevalence of 5.94%. Prevalence of the G6PD was studied in 125 vivax malaria cases confirmed by laboratory and among 604 persons determined as negative by laboratory testing. Positive cases consisted of patients with clinical manifestations of malaria and asymptomatic cases. The results of the investigations are presented in Table 2.

**Table 2:** Prevalence of G6PD among malaria patients and healthy persons, Azerbaijan, 1972 [67]

Groups	Total examined	G6PD deficient	%	OR	CI	p- value
+ve	125	11^a^	8.80	0.36	0.18–0.72	0.001
−ve	604	127	21.03			

^a^ 7 asymptomatic cases

The G6PD prevalence among malaria patients was lower than in non-infected persons (p < 0.05). Although statistically significant, the OR of 0.36 indicates that the G6PD deficiency does not prevent the contraction of infection. Even more epidemiologically important, however, is that among the 11 G6PD-deficient positive cases, 7 persons were asymptomatic cases, and only 4 positive cases were with clinical manifestations of the disease, suggesting that the G6PD deficiency played some role in the phenomenon of asymptomatic infection [67].

The results of the studies in Azerbaijan agree with those obtained in other malaria-endemic areas. Studies in the Brazilian Amazon (Manaus) among local populations demonstrated a significant protection of vivax malaria in the G6PD-deficient men enrolled in these studies, independently of their age [68]. Extension of research in the same areas corroborated the results and inferences of previous studies and revealed that the G6PD-deficient individuals with vivax malaria were less likely to report the occurrence of malaria episodes due to the protective effect related to the enzyme activity [69, 70].

Results of studies in the areas bordering Thailand-Myanmar showed no significant difference in the malaria positivity rate when comparing G6PD normal and deficient subjects. However, although not significant, parasite densities were a few times lower in the presence of G6PD deficiency compared to densities in normal subjects for *P. falciparum* and *P. vivax* [71].

Another aspect of the problem was recently discussed by Baird [72]. It appears that the problem of G6PD deficiency excludes many people from safe and effective treatment at the latent stage of vivax malaria, such as the G6PD-deficient persons, pregnant and lactating women, young infants, people with chronic hepatic problems, and a few others. As a result, a large fraction of the population constitutes a persistent source of infection in the community, both with clinical manifestations and asymptomatic carriers. A good illustration of such a situation is malaria incidence among excluded persons from mass drug administration with primaquine in the Democratic People Republic of Korea (Table 3).

**Table 3:** Malaria among people excluded from the mass drug administration with primaquine, Democratic People Republic of Korea, 2002–2003 [15, 73]

County	Target population	Total excluded	% excluded	Cases among excluded	Incidence
Panmum	30,000	2100	7	18	8.6
Hwanju	74,030	10,000	13.5	147	14.7
Anbyon	40,970	4978	12.1	123	24.7
Sonchon	54,470	6793	12.5	45	6.6
Kangnam	33,030	1950	5.9	14	7.2
Suchon	86,600	10,133	11.7	114	11.2
Hwanhu	2263	542	23.9	18	33.2
TOTAL	320,763	36,495	11.38	479	13.1

## Probable role of β-thalassaemia in *Plasmodium vivax* asymptomatic malaria

The mechanisms by which the thalassaemia protects against malaria are not known with any certainty [74]. Reduced parasite growth occurs in β-thalassaemia cells, particularly when exposed to oxidant stress. Infected cells from β-thalassaemia subjects show enhanced antigen expression at the surface of the infected red cells, possibly leading to enhanced immune clearance and further to mild attacks and an asymptomatic course of infection [74].

The highest prevalence of β-thalassaemia was found in the territory of the former USSR in Azerbaijan, where its prevalence was, on average, 8.67%, ranging from 9.28% in the lowlands to 10.16% in the foothills; both populations compose 70% of the total population of the country. The highest prevalence of 16.8% was found in one district in the foothills. Very low prevalence was established in the mountains (1000–2000 m asl): 1.30%, with 0% prevalence in the high mountains. The distribution of β-thalassaemia almost ideally coincided with the distribution of malaria in the past and in the present. Importantly, a strong correlation in the analogous geographical distribution of the G6PD deficiency had also been marked. Another important finding was that, on making a comparison with the occurrence of different forms of malaria, areas with high concentrations of the gene for β-thalassaemia did not correspond with the occurrence of *P. falciparum* in the past; rather, they corresponded with the occurrence of *P. vivax* [75, 76]. Thus, coexistence of the G6PD deficiency and β-thalassaemia among the local population in Azerbaijan might contribute to the phenomenon of asymptomatic malaria in the country, which may account for the difficulty in eliminating malaria in this country.

## Asymptomatic malaria due to mixed parasitic diseases

A detailed review of published papers on the interactions between worms and malaria has led to the conclusion that, at present, no clear picture exists as to the outcome of this interaction [77]. Nevertheless, some worms, much more so than others, demonstrate protective properties against malaria and its severe manifestations. *Ascaris lumbricoides* is one such parasite, whereas hookworms seem to increase malaria incidence and manifestations [78]. Important epidemiological evidence transcribed from these publications indicate that nematode worms in malaria patients are capable of reducing some symptoms (e.g., body temperature) of the disease, thus precluding patients seeking treatment and thus contributing to the malaria infection remaining asymptomatic [78–80]. This inference was recently further confirmed by the results of a prospective cohort study in Mali. Co-infection with *Schistosoma haematobium* and *P. falciparum* was significantly associated with a reduced risk of febrile malaria in long-term asymptomatic carriers of *P. falciparum* [81].

## Association of malaria with HIV infection

Malaria and HIV are major global health problems, particularly in the countries south of Sahara. Both infections share extensive epidemiological overlap, co-infecting large numbers of people in many endemic countries of the world. HIV-1 infection has long been associated with an increased frequency of clinical malaria and parasitaemia. This association became even more pronounced with advancing immunosuppression [82]. Furthermore, HIV co-infection is not only is associated with increased disease severity but also with malaria mortality in an area of stable malaria transmission with an overwhelming preponderance of *P. falciparum* [83]. However, recent studies in Nigeria have revealed that, under certain situations, for example, in children under 5 years of age, asymptomatic malaria was more common in HIV-positive children [84].

The situation in areas with relatively low levels of malaria transmission appeared somewhat different. The results of studies in southern India showed that *P. vivax* accounted for a majority of the infections (60%), followed by *P. falciparum* (27%) and mixed infections (13%). *Plasmodium falciparum* infection was more than sixfold higher among HIV-positive individuals compared with their counterparts infected with *P. vivax*, thus suggesting that a number of *P. vivax* cases could be asymptomatic cases [85].

Results of a comparative cross-sectional study in Oromi, Ethiopia, known for local transmission of both *P. vivax* and *P. falciparum*, documented lower malaria prevalence among HIV-seropositive individuals who came for routine follow-up. Clinical symptoms of malaria were more pronounced among HIV-seronegative than HIV-seropositive patients [86]. On the whole, in epidemiological settings with high malaria transmission (*P. falciparum*-predominant malaria), asymptomatic malaria is associated with HIV-seronegative infections. In areas with moderate and low-level malaria transmission, *P. vivax* asymptomatic cases are related to HIV-positive infection.

## Implications for the diagnosis of asymptomatic vivax malaria

Several recently conducted studies in areas of low malaria transmission in Ethiopia demonstrated the presence of asymptomatic malaria in both *P. vivax* and *P. falciparum*, which could not be detected either by microscopy or RDT [86–88]. The results of these studies showed that malaria infections were not detected by microscopy despite more than 5% prevalence detected by nPCR (nested polymerase chain reaction: 5.2% for *P. falciparum* and 4.3% for *P. vivax* [86–88]. The inadequate sensitivity of microscopy and RDT to detect substantial sub-microscopic parasitaemia undoubtedly would affect malaria control/elimination plans of national malaria programmes aiming to eliminate malaria in the nearest future. Thus, PCR and its modifications (including nPCR, real-time PCR) have sufficient sensitivity to detect a higher number of infected subjects with low and sub-microscopic parasite densities than RDT and microscopy, potentially accelerating the achievement of malaria elimination [89].

## Conclusions

Review of published data on the peculiarities of *P. vivax* epidemiology during the various stages of malaria elimination programmes at present and malaria eradication programmes in the past, particularly in the republics of the former USSR, revealed the southward extension of vivax malaria with long incubation, an increase of severe cases, a reduced response to certain anti-malarials, and the appearance of a large number of asymptomatic cases. One of the major problems facing malaria elimination programmes at its final stage is the difficulty in detection of cases under conditions of drastic reduction of intensity of malaria transmission. Retrospective analysis of published data in the former USSR and in other countries on the same subject have revealed various factors contributing to the problem, due to a mild course of infection, reduced level of clinical manifestations of the disease and low density of malaria parasites under a microscope. Such situations inevitably led to the occurrence of asymptomatic malaria cases, the epidemiological role of which is still underestimated in many situations. Additional contributing factors might be the presence of genetic disorders such as G6PD deficiency and β -thalassaemia, and probably some others. Malaria in association with other parasitic diseases represents another group of situations, potentially contributing to the development of asymptomatic malaria. Technical staff of national malaria elimination programmes, as well as personnel of health services, engaged in the implementation of anti-malaria activities should be aware of the problem of the detection of asymptomatic malaria. This could be done through the orientation training of the staff. Deployment of various approaches towards strengthening case detection mechanisms should be considered/introduced, including proactive case detection, use of PCR, nested PCR and other mechanisms. The need exists to further develop suitable methods of field detection of malaria asymptomatic cases.

## Additional files

**Additional file 1.** Malaria control and elimination in the former USSR.

**Additional file 2.** *Plasmodium vivax* fulminant malaria in the former USSR.

## Abbreviations

PCD: passive case detection
PCR: polymerase chain reaction
USSR: Union of Soviet Socialist Republics
WHO: World Health Organization
