# Supplementary material for: On the epidemiology of Plasmodium vivax malaria: past and present with special reference to the former USSR
Source: Malar J. 2018 Oct 4;17:346. doi: 10.1186/s12936-018-2495-y (PMC6172834; doi:10.1186/s12936-018-2495-y)
Supplement: Supplementary file 1 — Additional file 1. Malaria control and elimination in the former USSR. [file 12936_2018_2495_MOESM1_ESM.docx]

**ADDDITIONAL FILE 1.**

**Malaria Control and Elimination in the former USSR**

Historical periods

There are 5 historical periods of malaria control on the territory of Imperial Russia (1900-1917) and the former USSR (1918-1991).

*First period covers the years 1900 through 1918*. Although malaria was considered as one of the major health problems in the Imperial Russia, attempts to control devastating infection were very few. According to various estimates, total of primary cases of malaria annually was not less than 5 million cases (Favr, 1903). Most affected population was in the territory of Caucasia, Transcaucasia, Middle Asia, in the Basin of Volga River, European part of Russia, Siberia. Large scale malaria epidemics were a frequent picture in the country. Malaria mortality during epidemic was very high. For example, more than 35% of total population died due to malaria in one of the vilayats of Tashkent region in epidemic of 1894.

*Second period- beginning of malaria control in the USSR – 1920-*1935.

Following the initiative of EI Martzinovski, Central State Institute of protozoa infections and chemotherapy under the Health Ministry of the USSR was established in 1920 and a few similar institutions were set in other Republics of the Union. In the following years a network of antimalaria stations was established across the country. By the year 1930, there were total of 231 stations in the USSR. The scope of the activities of the stations was the treatment of malaria patients by quinine, imported from abroad. The impact of treatment alone was rather limited and could not prevent the occurrence of malaria epidemics during 1933-1935 (figure 6).

**Figure 6. Malaria in Russia and the former USSR (1900-1963)**

*Third period – Country-scale organized malaria control (1935-1953).*

The most important development was an inclusion of malaria control into the National Plan of Development of the USSR in 1934. This was done in consideration of enormous negative impact of malaria on the health status of population and on economy of the country. During 1935-1937, malaria incidence was exceeding 1 million cases each in the Russian Federation and Ukraine, and more than 100 000 cases annually in Kazakhstan, Tajikistan, Uzbekistan, Armenia, Azerbaijan, Georgia. (Table 1).

To meet malaria challenge, the number of antimalarial stations was remarkably increased, accompanied by capacity building. Training of malariologists both at the high education and auxiliary levels dramatically increased. In 1935, Malariology Chair was established at the Central Academy of Post Diploma Education in Moscow, responsible for training of malariologists, entomologists, laboratory specialists, sanitary engineers and alike. Very important development was the establishment of “Acriquine” factory manufacturing anti malaria drugs on a national scale. To facilitate the implementation of antimalaria measures, the Government of the USSR in 1934 issued an Order which made responsible the local Soviet and Party institutions for its accomplishment. This action led to the mobilization of large groups of population to participate in the implementation of antimalaria measures. As a result, by 1940, malaria burden was reduced almost thrice as compared with 1934. This trend was however arrested by the Second World War (1939-1945) and during immediately after the War. It came back and further down with the introduction of DDT as a major anti vector intervention on a large scale, and starting from 1950, total number of cases came down to less than one million (Figure 6). By the end of the third period, malaria incidence was less than 10 per 10 thousand population. It was accepted in the country as an indicator of malaria elimination as a mass disease and allowed to embark on practical elimination of malaria.

*Fourth period – Elimination of malaria (1954-the 1960s).*

Large scale use of DDT in conjunction with other antimalaria interventions brought down malaria incidence to the level of total of 21 1 indigenous cases of malaria in 1963 and it was considered that the disease was practically eliminated on the territory of the USSR.

Table 1. P.vivax malaria in the Republiсs of the former USSR (1934-1963) [Sergiev et al, 1968)

| Republic | 1934 | 1940 | 1946 | 1950 | 1956 | 1960 | 1963 |
| --- | --- | --- | --- | --- | --- | --- | --- |
| Russia | 5.3 m | 1.9 m | 1.6 m | 0.36 m | 5 591 | 122 | 76 |
| Ukraine | 1.9 m | 0.24m | 0.35m | 0.045 m | 184 | 17 | 9 |
| Belorussia | 0.009m | 0.02m | 0.23 m | 0.02 m | 46 | 1 | 0 |
| Uzbekistan | 0.32 m | 0.21m | 0.35m | 0.12m | 645 | 12 | 22 |
| Kazakhstan | NA | 0.10m | 0.23m | 0.006m | 1 103 | 44 | 33 |
| Georgia | 0.49m | 0.10m | 0.078m | 17403 | 808 | 15 | 12 |
| Azerbaijan | 0.53m | 0.23m | 0.24m | 0.061m | 3 071 | 85 | 31 |
| Lithuania | NA | NA | 1 632 | 239 | 10 | 0 | 0 |
| Moldavia | NA | 0.059m | 0.064m | 0.031m | 51 | 1 | 0 |
| Latvia | NA | NA | 1 590 | 406 | 4 | 0 | 3 |
| Kyrgyzstan | NA | 0.018m | 0.052m | 0.01 m | 69 | 0 | 1 |
| Tajikistan | 0.10 m | 0.072m | 0.08m | 0.027m | 1 305 | 61 | 21 |
| Armenia | 0.189m | 0,053m | 0.061m | 0.014m | 68 | 4 | 0 |
| Turkmenistan | 0.107m | 0.027m | 0.029m | 7 398 | 58 | 6 | 1 |
| Estonia | NA | NA | 527 | 168 | 1 | 0 | 2 |
| TOTAL | 9.48m | 3.18m | 3.36m | 0.78m | 13 014 | 368 | 211 |

*Fifth period – maintenance of results achieved (1963-1991- disintegration of the USSR)*

Malaria Surveillance Program in the USSR was introduced following achieved practical elimination of malaria in the country in 1963. During this period, only sporadic cases of *P.vivax* malaria were registered in the residual foci in Azerbaijan and Tajikistan, necessitating implementation of vector control activities, detection and treatment of cases among local population and immigrants. During the 1970-80s, a few outbreaks of *P.vivax* malaria were registered in Azerbaijan and Tajikistan and local malaria transmission was resumed on the territory of these Republics. Proliferation of *P.vivax* into the territories of neighboring republics took place at the beginning of the 1990s coinciding with disintegration of the USSR. Containment of these outbreaks occurred during the 1990s-beginning XXI century. At present, local transmission in Azerbaijan, Armenia, Kazakhstan, Kyrgyzstan, Georgia, Tajikistan, Turkmenistan, Uzbekistan was interrupted. Armenia, Kazakhstan, Kyrgyzstan and Turkmenistan were certified by the World Health Organization as countries free of malaria.
