# Supplementary material for: On the epidemiology of Plasmodium vivax malaria: past and present with special reference to the former USSR
Source: Malar J. 2018 Oct 4;17:346. doi: 10.1186/s12936-018-2495-y (PMC6172834; doi:10.1186/s12936-018-2495-y)
Supplement: Supplementary file 2 — Additional file 2. Plasmodium vivax fulminant malaria in the former USSR. [file 12936_2018_2495_MOESM2_ESM.docx]

**Additional File 2.**

**P.vivax fulminant malaria in the former USSR**

First reliable data (based on laboratory and postmortem sections) on cases of death due to so called “ fulminant” P. vivax malaria (malaria tertian siderans) on the territory of the ex-USSR relate to the 1920s-1930s (Bistrov, 1927; 1940;1945). A series of clinical descriptions of the disease appeared during the 1939s-1950s (Alperovich et al., 1937; Lopatin, 1937’, 1945; Gontaeva, 1942; 1945;1953, Tareev, 1945).

Geographical confinement of P.vivax fulminant malaria is shown in Fig 1. (“Geographical distribution of P.vivax fulminant cases in the former USSR, 1935-1947”). (Figure 4).

It can be seen that cases of fulminant P.vivax malaria were reported in 3 republics of the ex-USSR namely Russian Federation, Republic of Uzbekistan, and Republic of Kyrgizstan. Infection was overwhelmingly confined to the territory of Russian Federation, particularly to the Basin of Volga River.

One of the peculariaties of this form was its preponderance to the children between 4-14 years of age. The peak of fulminant cases occurred during the spring months in patients with primary manifestations of the disease indicating its belonging to P.vivax with long incubation. Some cases occurred in persons acquiring infection during the last year.

Epidemiological investigation of 104 fulminant cases of P.vivax in the city of Kuibishev (now Samara) in 1935-1945 revealed that they were registered during the whole calendar year both among the children and adults. The highest number cases (death) was observed in August-September, following the seasonal pattern of malaria for that site. P.vivax was presented both with short- and long incubation.

There was a well pronounced seasonal difference between cases among the adults and children. The majority of cases in children occurred in the first half of the year (62.3%), e.g . the occurrence of relapses of short incubation and the first cases of long incubation, while among the adults the majority of cases occurred in the second half of the year (91.6%), e.g. manifestations of fresh cases of short incubation. It was also observed that the level of incidence of fulminant cases among children and adults varied from year to year. In 1940, 1941, 1942 and especially in 1945, the incidence among the children was greatly exceeded that among the adults. Opposite situation was observed in 1943-1944. The majority of cases in children occurred in the group of 2-5 years of age. It was also found that the death due to fulminant malaria was overwhelmingly occurring in patients who did not timely receive appropriate treatment.

Analysis of clinical manifestations of 900 cases of P.vivax fulminant cases on the territory of Russian Federation by Prokopenko (1959) showed that primary clinical signs resembled its typical for P.vivax malaria attack. However heavy headache appeared very quickly accompanied by nausea and vomiting. Next day the child would be feeling much better, could be playing and even attending the school. However the appearance of the second attack would be accompanied by cerebral symptoms like unbearable headache with such intensity that child would be crying and vomit. Sleepiness, convulsions, unconsciousness, Chain-Stocks breathing joins very quickly and death would occur within 2-3 hours, sometime after 5-6 hours.

While alive, malaria parasites in the thick blood smear of a patient would be found in a very low numbers. A few malaria parasites would be found in the brain’s capillaries.

It was thought that probable cause of death due to P.vivax malaria was anoxia of brain due to acute allergic swelling of the latter (Kassirski et al, 1974{. Experience showed that to prevent a fatal exit, there was a need to carry out immediate anti malaria treatment in conjunction with the deployment of other specific medicaments. ((Sergiev et al, 1968).

REFERENCES

1. *Bistrov P.V.(*1927). On malaria mortality in P.vivax malaria . J. microbiology, pathology and infectious diseases, v.IV, 3:224-244 (in Russian)
2. *Bistrov V.P.* (1940).Pathological anatomy of acute vivax malaria. Archives of Pathological Anatomy and Pathological Phisiology. v. IV. 1-2: 111-120 (in Russian)
3. *Bistrov P.V*. (1945). Fulminant vivax malaria (based on postmortem sections in Tambov). Med. Parasitol. Parasit.Dis., v. XIV, 2:21-25 (in Russian)
4. *Alperovich Ya.A and Gelfer G.A.* (1937). Diseases of internal organs due to malaria. In: Transactions of inter-regional conference of therapeutists of Gorky city., 328-348 (in Russian)
5. *Lopatin G.M*. (1945). On deaths due to malaria in children. Pediatria, 5, 24-25 (in Russian)
6. *Gontaeva A.A. (1945*). Recognition and treatment of severe malaria. Sovetskaya Meditzina, 31, p.25 (in Russian)
7. *Tareev et al.(*1943). Fulminant vivax malaria. Sovetskaya Melitzina, 4, 12-14 (in Russian)
8. *Prokopenro LI* (1959). *“*On epidemiology of fulminant P.vivax malaria”, in: Transactions of the Moscow Martzinovski Institute of Medical Parasitology and Tropical Medicine, Editors: V.N.Beklemishev and M.G.Rashina, Moscow, pp 80-102 {in Russian).
9. *Sergiev PG et al (1968)* Malaria*.* In: Manual of Microbiology, Medicina, Moscow, v.IX: 61-62 (In Russian)
10. *Kassirski IA, Plotnikov NN, Tokarev YN, Lysenko AY. (1974).* Manual of Tropical Diseases, Medicina, Moscow: 423 p(In Russian)
